# The *PTPN22* Locus and Rheumatoid Arthritis: No Evidence for an Effect on Risk Independent of Arg620Trp

**DOI:** 10.1371/journal.pone.0013544

**Published:** 2010-10-21

**Authors:** Wan R. Wan Taib, Deborah J. Smyth, Marilyn E. Merriman, Nicola Dalbeth, Peter J. Gow, Andrew A. Harrison, John Highton, Peter B. B. Jones, Lisa Stamp, Sophia Steer, John A. Todd, Tony R. Merriman

**Affiliations:** 1 Department of Biochemistry, University of Otago, Dunedin, New Zealand; 2 Juvenile Diabetes Research Foundation/Wellcome Trust Diabetes and Inflammation Laboratory, Department of Medical Genetics, Cambridge Institute for Medical Research, Addenbrooke's Hospital, Cambridge, United Kingdom; 3 Department of Medicine, University of Auckland, Auckland, New Zealand; 4 Department of Rheumatology, Middlemore Hospital, Auckland, New Zealand; 5 Department of Medicine, University of Otago, Wellington, New Zealand; 6 Department of Medicine, University of Otago, Dunedin, New Zealand; 7 Department of Medicine, University of Otago, Christchurch, New Zealand; 8 Department of Rheumatology, Kings College London School of Medicine at Guy's, King's and St. Thomas', London, United Kingdom; Leiden University Medical Center, Netherlands

## Abstract

**Objectives:**

The Trp^620^ allotype of PTPN22 confers susceptibility to rheumatoid arthritis (RA) and certain other classical autoimmune diseases. There has been a report of other variants within the PTPN22 locus that alter risk of RA; protective haplotype ‘5’, haplotype group ‘6–10’ and susceptibility haplotype ‘4’, suggesting the possibility of other *PTPN22* variants involved in the pathogenesis of RA independent of R620W (*rs2476601*). Our aim was to further investigate this possibility.

**Methods:**

A total of 4,460 RA cases and 4,481 controls, all European, were analysed. Single nucleotide polymorphisms *rs3789607*, *rs12144309*, *rs3811021* and *rs12566340* were genotyped over New Zealand (NZ) and UK samples. Publically-available Wellcome Trust Case Control Consortium (WTCCC) genotype data were used.

**Results:**

The protective effect of haplotype 5 was confirmed (*rs3789607*; (OR = 0.91, *P* = 0.016), and a second protective effect (possibly of haplotype 6) was observed (*rs12144309*; OR = 0.90, *P* = 0.021). The previously reported susceptibility effect of haplotype 4 was not replicated; instead a protective effect was observed (*rs3811021*; OR = 0.85, *P* = 1.4×10^−5^). Haplotypes defined by *rs3789607*, *rs12144309* and *rs3811021* coalesced with the major allele of *rs12566340* within the adjacent BFK (B-cell lymphoma 2 (BCL2) family kin) gene. We, therefore, tested *rs12566340* for association with RA conditional on *rs2476601*; there was no evidence for an independent effect at *rs12566340* (*P* = 0.76). Similarly, there was no evidence for an independent effect at *rs12566340* in type 1 diabetes (*P* = 0.85).

**Conclusions:**

We have no evidence for a common variant additional to *rs2476601* within the *PTPN22* locus that influences the risk of RA. Arg620Trp is almost certainly the single common causal variant.

## Introduction

Genome-wide association (GWA) scans have emphasised the importance of the *PTPN22* gene, encoding the phosphatase LYP, in susceptibility to rheumatoid arthritis (RA) in European and European-ancestry populations, with *HLA* and *PTPN22* locus SNPs dominating association at the genome-wide level.[1]–[3] The Arg620Trp variant, encoded by SNP rs2476601 (C>T), of the protein tyrosine phosphatase-22 (PTPN22) gene, is a prominent determinant of some autoimmune phenotypes, including RA. Strong association of the Trp620 variant has been repeatedly demonstrated to RA, type 1 diabetes (T1D), Graves' disease (GD) and systemic lupus erythematosus (SLE) in European populations.[4] The Trp620 allele is very rare in Asian populations.[5] The Trp620 effect seems to be restricted to autoimmune phenotypes in which a defined auto-reactivity is evidenced by specific autoantibodies. The LYP protein is part of a complex down-regulating signal from the activated T-cell receptor (TCR), with biochemical studies suggesting that PTPN22 inhibits T-cell activation through dephosphorylation of the LCK and ZAP-70 kinases.[6] The Trp^620^ allotype of LYP is unable to interact with CSK, and generates a more active phosphatase that is more effective in inhibiting TCR signalling than the Arg^620^ allotype.[7] In the thymus this might result in the positive selection of autoreactive T-cells that would otherwise be deleted or, in the periphery, reduced TCR signalling in T-regulatory cells resulting in reduced regulation of autoreactive T-cells. The Trp^620^ allotype also impairs signalling from the B-cell receptor.[8]

The PTPN22 gene maps to chromosome 1p13.2 within a haplotype block of conserved linkage disequilibrium (LD) spanning over 300 kb.[1] To investigate the possibility that further genetic variants within *PTPN22* have a role in RA, coding regions within the gene were resequenced in USA people of white ethnic group-European ancestry and ten common haplotypes were tested for association with RA.[9] This analysis confirmed the predominant role in disease susceptibility conferred by the haplotype tagged by the Trp620 allele (minor allele of rs2476601: C>T) (haplotype ‘2’). The analysis also provided evidence for a second haplotype that increases risk of RA (haplotype ‘4’) independent of the Arg620Trp variant. However this effect was not replicated in Norwegian, Dutch or UK sample sets.[10]–[12] Interestingly, some variants in *PTPN22* provided evidence for a protective association with RA. A haplotype uniquely defined by SNP rs12760457 was associated with protection from RA (haplotype ‘5’),[9] although this association was not replicated in UK and Dutch RA sample sets.[10], [11] Whilst this association was evident in T1D, it was not independent of the Arg620Trp effect.[13] In RA, analysis of inter-marker LD and extent of association of SNPs within the extended *PTPN22* haplotype block in a GWA scan of pooled genomic DNA samples from New Zealand and the UK suggested the presence of a second susceptibility determinant that was not explained by LD with the Trp620 variant, and perhaps related to the protective ‘haplotype 5’ identified by Carlton et al.[1], [9] Collectively the previous reports [1], [9] provided evidence for a second (protective) common RA risk allele or haplotype within the *PTPN22* locus, that may map outside of the PTPN22 gene. Indeed association of a rare functional *PTPN22* variant (Arg263Gln, rs33996649:G>A, minor allele frequency  = 2.6% in Caucasian) with SLE and RA has been reported, with the A allele conferring a protective effect independent of *rs2476601*.[14], [15]

Here, our aim was to further investigate the possibility of allelic heterogeneity at *PTPN22* in RA, focusing on the possibility of the existence of a common RA-protective haplotype independent of Arg620Trp. Here, despite replicating the protective association of haplotype ‘5’ with RA, there was no evidence that this association was independent of *rs2477601*.

## Results

The study of Carlton et al [9] first reported association of haplotype ‘5’ with RA (*P* = 1.5×10^−5^) in 1122 cases and 1767 controls. In an attempt to replicate the association of haplotype 5 with RA, a haplotype 5-defining SNP (*rs3789607*) was genotyped over the separate NZ and UK (London) RA case-control sample sets (Table 1). These data were combined with that of Wesoly et al [11] and with imputed data from the publically-available Wellcome Trust Case Control Consortium (WTCCC) RA genome-wide association (GWA) scan sample set.[2] The data of Hinks et al [10] were not used as they overlap with that of the WTCCC. The combined analysis of the UK, Dutch and NZ data provided independent evidence supporting protective association of haplotype 5 with RA (M-H pooled OR = 0.91 [0.85–0.98], *P* = 0.016).

**Table 1:** Analysis of association of *PTPN22* ‘haplotype 5’ (*rs3789607*: T>C) with rheumatoid arthritis.

	Genotype^1^					
Cohort	T/T	T/C	C/C	C Allele^2^	OR [95% CI]^ 4^	Allelic *P*
Carlton Set 1	257 (0.552)189 (0.408)	173 (0.371)220 (0.475)	36 (0.077)54 (0.117)	245 (0.263)328 (0.354)	0.65 [0.53–0.79]	2×10^−5^
Carlton Set 2	350 (0.534)659 (0.505)	274 (0.418)512 (0.393)	32 (0.049)133 (0.102)	338 (0.258)778 (0.298)	0.82 [0.70–0.95]	0.008
**Combined Carlton**	**607 (0.541)** **848 (0.480)**	**447 (0.398)** **732 (0.414)**	**68 (0.061)** **187 (0.106)**	**583 (0.260)** **1106 (0.313)**	**0.77 [0.68**–**0.87]**	**1.5×10^−5^**
Wesoly	357 (0.536)148 (0.523)	269 (0.404)119 (0.421)	40 (0.060)16 (0.056)	349 (0.262)151 (0.267)	0.98 [0.78–1.23]	0.83
WTCCC^3^	933 (0.502)1434 (0.488)	789 (0.424)1253 (0.426)	138 (0.074)251 (0.085)	1065 (0.286)1755 (0.299)	0.94 [0.86–1.03]	0.19
NZ	452 (0.526)263 (0.466)	344 (0.400)248 (0.440)	64 (0.074)53 (0.094)	472 (0.274)354 (0.314)	0.83 [0.70–0.97]	0.023
UK London	129 (0.549)86 (0.521)	94 (0.400)64 (0.388)	12 (0.051)15 (0.091)	118 (0.251)94 (0.285)	0.84 [0.61–1.16]	0.29
**Combined other**	**1871 (0.517)** **1931 (0.487)**	**1496 (0.413)** **1684 (0.427)**	**254 (0.070)** **335 (0.085)**	**2004 (0.277)** **2354 (0.298)**	**0.90 [0.84–0.97]**	**0.003**
***TOTAL***	***2478 (0.523)*** ***2779 (0.486)***	***1943 (0.410)*** ***2416 (0.423)***	***322 (0.068)*** ***522 (0.091)***	***2587 (0.273)*** ***3460 (0.303)***	***0.86 [0.81–0.92]***	***1.7×10^−6^***

1 Cases top line, controls bottom line. The Carlton Set 2 cases and controls, the combined other cases and total cases deviated mildly from HWE (*P* = 0.02, 0.03, 0.05 and 0.02, respectively).

2 Allele 2; number of chromosomes (frequency).

3 Genotype data from *rs17274634* were used (r^2^ = 1 with *rs3789607* in CEPH CEU (www.hapmap.org)).

4 The Mantel-Haenszel pooled OR = 0.87 [0.82–0.93], *P* = 7.5×10^−6^; Breslow-Day test for heterogeneity *P* = 0.083. The Mantel-Haenszel pooled OR excluding Carlton et al data was 0.91 [0.85–0.98], *P* = 0.016; Breslow-Day *P* = 0.50.

**Figure 1:**
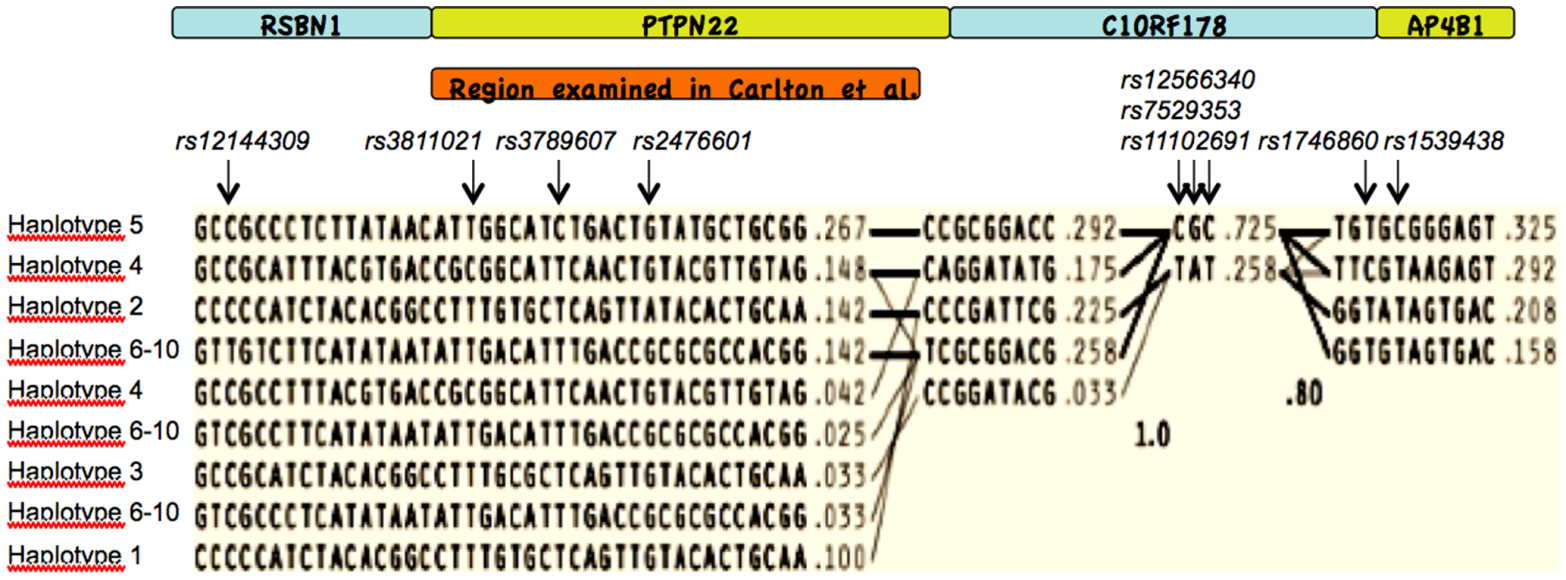
Haplotype structure of structure of a portion of the *PTPN22* haplotype block. The figure was generated by Haploview using Phase 2 CEPH CEU HapMap data downloaded from www.hapmap.org, with the boundaries being *rs4145859* (114.312 Mb) and *rs10745340* (114.437 Mb).

Given some evidence for an RA protective effect conferred by haplotype 6 in the Carlton et al [9] sample sets (*P* = 0.15 in sample set 1; *P* = 5×10^−5^ in sample set 2; combined one-tailed *P* by Fisher's method <0.0001), we further investigated this finding in the NZ, UK (London) and WTCCC cohorts. Haplotypes 6–10 can be distinguished from haplotypes 1–5 by *rs1217414.* [9] Four SNPs, none of which have been genotyped in CEPH CEU by HapMap (www.hapmap.org; release 23a), are required to distinguish haplotype 6 from haplotypes 7–10, meaning it would not be possible to use imputation to test these SNPs for association in the WTCCC data. Instead we used *rs12144309* to investigate further association of the haplotype 6–10 group with RA - *rs12144309*, which maps outside of the region studied by Carlton et al [9], identifies one major haplotype within the 6–10 group (Figure 1). In the CEU samples, *rs12144309* tagged one haplotype of frequency 14.2%, with the remaining 6–10 haplotypes constituting 5.8%. *rs12144309* was tested for association with RA in the WTCCC dataset by use of the publically-available imputation data, and by genotyping across the NZ and UK (London) sample sets (Table 2). The resultant data (M-H pooled OR = 0.90 [0.82–0.98], *P* = 0.021) confirmed association of a protective haplotype within the haplotype 6–10 group with RA. However it is not possible in this case to ascribe this association to the same haplotype identified by Carlton et al. (haplotype 6).[9] These data were unable to be combined with that of Wesoly et al [11]; however, in their sample *P* for association of haplotype 6 with RA was 0.48 (frequency of 10.7% in cases, 11.9% in controls).

**Table 2:** Analysis of association of *PTPN22* ‘haplotype 6–10’ group (*rs12144309*: C>T) with rheumatoid arthritis.

	Genotype^1^					
Cohort	C/C	C/T	T/T	T Allele^3^	OR [95% CI]^ 2^	Allelic *P*
WTCCC^2^	1230 (0.662)1857 (0.633)	558 (0.300)958 (0.326)	70 (0.038)120 (0.041)	698 (0.188)1198 (0.204)	0.90 [0.81–1.00]	0.052
NZ	612 (0.715)387 (0.689)	231 (0.270)168 (0.299)	13 (0.015)7 (0.012)	257 (0.150)182 (0.162)	0.91 [0.74–1.12]	0.40
UK London	172 (0.720)114 (0.667)	60 (0.251)52 (0.304)	7 (0.029)5 (0.029)	74 (0.155)62 (0.181)	0.83 [0.57–1.20]	0.31
***TOTAL***	***2014 (0.682)*** ***2358 (0.643)***	***849 (0.288)*** ***1178 (0.321)***	***90 (0.030)*** ***132 (0.036)***	***1029 (0.174)*** ***1422 (0.194)***	***0.88 [0.80–0.96]***	***0.004***

1. Cases top line, controls bottom line. The NZ controls deviated mildly from HWE (*P* = 0.02).

2. Imputed genotypes were taken from www.wtccc.org.uk.

3. The Mantel-Haenszel combined OR = 0.90 [0.82–0.98], *P* = 0.021. The Breslow-Day test for heterogeneity *P* = 0.90.

Carlton et al [9] reported positive association of haplotype ‘4’ (tagged by *rs3811021*) with RA (combined OR = 1.20, *P* = 0.009). We genotyped this SNP in the NZ and UK London cohorts and analysed association with RA, with the inclusion of data from the WTCCC [2], Wesoly et al [11] and Viken et al [12] studies (Table 3). Meta-analysis by the Mantel-Haenszel method of all the available data demonstrated heterogeneity (Breslow-Day *P*<0.001; OR  = 0.85 [0.72–1.01], *P* = 0.071), with separate analysis after exclusion of the Carlton et al [9] data demonstrating a protective effect mediated by haplotype ‘4’ in the sample sets combined here (Breslow-Day *P* = 0.12; M-H OR = 0.85 [0.78–0.91], *P* = 1.4×10^−5^).

**Table 3:** Analysis of association of *PTPN22* ‘haplotype 4’ (*rs3811021*: A>G) with rheumatoid arthritis.

	Genotype^1^					
Cohort	A/A	A/G	G/G	G Allele	OR [95% CI]^3^	Allelic *P*
Carlton Set 1	287 (0.619)323 (0.699)	162 (0.349)128 (0.277)	15 (0.032)11 (0.024)	192 (0.207)150 (0.162)	1.35 [1.06–1.70]	0.014
Carlton Set 2	425 (0.645)883 (0.670)	197 (0.299)383 (0.291)	37 (0.056)51 (0.039)	271 (0.206)485 (0.184)	1.15 [0.97–1.35]	0.11
**Combined Carlton**	**712 (0.634)** **1206 (0.678)**	**359 (0.320)** **511 (0.287)**	**52 (0.046)** **62 (0.035)**	**463 (0.206)** **635 (0.178)**	**1.20 [1.05–1.37]**	**8.8×10^−3^**
Wesoly	461 (0.693)177 (0.623)	188 (0.283)97 (0.341)	16 (0.024)10 (0.035)	220 (0.165)117 (0.206)	0.76 [0.59–0.99]	0.034
Viken	563 (0.657)337 (0.609)	261 (0.305)187 (0.338)	33 (0.039)29 (0.052)	327 (0.191)245 (0.222)	0.83 [0.69–1.00]	0.047
WTCCC^2^	1244 (0.669)1900 (0.648)	558 (0.300)917 (0.313)	57 (0.031)117 (0.040)	672 (0.181)1151 (0.196)	0.90 [0.81–1.01]	0.061
NZ	645 (0.752)389 (0.697)	201 (0.234)159 (0.285)	12 (0.014)10 (0.018)	225 (0.131)179 (0.160)	0.79 [0.63–0.97]	0.030
UK London	179 (0.810)105 (0.691)	42 (0.190)43 (0.283)	0 (0.000)4 (0.026)	42 (0.095)51 (0.168)	0.52 [0.34–0.81]	0.003
**Combined other**	**3092 (0.693)** **2908 (0.649)**	**1250 (0.280)** **1403 (0.313)**	**118 (0.026)** **170 (0.038)**	**1486 (0.167)** **1743 (0.194)**	**0.83 [0.77–0.89]**	**1.2×10^−6^**
***TOTAL***	***3804 (0.681)*** ***4114 (0.657)***	***1609 (0.288)*** ***1914 (0.306)***	***170 (0.030)*** ***232 (0.037)***	***1949 (0.175)*** ***2378 (0.190)***	***0.90 [0.84–0.96]***	***2.2×10^−3^***

1 Cases top line, controls bottom line.

2 Imputed genotypes were taken from www.wtccc.org.uk.

3 The Mantel-Haenszel combined OR  = 0.85 [0.72–1.01], *P* = 0.071. The Breslow-Day test for heterogeneity *P*<0.001.

Thus, within the PTPN22 gene there was replicated evidence for two protective effects, as defined by haplotype 5 and within the haplotype 6–10 group, and the protective effect we observed from haplotype 4. Analysis of association at the three tagging SNPs (*rs3789607*, *rs3811021* and *rs12144309*) conditional on genotype at *rs2476601* in the combined WTCCC and NZ samples, revealed no evidence for association with RA at any of the SNPs independent of *rs2476601* (all *P*>0.05). Haplotypes 4,5 and 6–10 are unrelated to each other over the physical boundaries of the PTPN22 gene [9] (Figure 1), and all contain Arg620. We invoked an alternative explanation to account for the observed protective effects in RA observed by us and Carlton et al [9]; that the protective effects are caused by a single allele in the *PTPN22* haplotype block, but outside of the region encompassing *PTPN22* that was assessed by Carlton et al [9] (Figure 1), and not due to the Arg620 allele. The LD relationship of haplotypes 4, 5 and 6–10 with markers in the extended *PTPN22* haplotype block was examined; all these haplotypes coalesce with the major allele of a group of markers exhibiting very strong inter-marker LD (Figure 1; r^2^>0.90; *rs12566340*, *rs7529353*, *rs11102691*). These markers all map within the 3′ untranslated region (UTR) of the BFK (B-cell lymphoma 2 (BCL2) family kin) gene (*C1orf178*). We hypothesized that this group of markers was responsible for the protective effects of haplotypes 4, 5 and 6–10 within the *PTPN22* region. *Rs12566340* was genotyped in the NZ RA sample set and imputed in the WTCCC RA sample set. Conditional analysis revealed weak evidence for association of *rs12566340* independent of *rs2476601* in the separate NZ and WTCCC datasets (*P* = 0.036 and 0.033, respectively). Two-marker *rs2476601-rs12566340* haplotypes were then estimated. In the NZ sample set comparison of the risk conferred by the C-C haplotype to that conferred by the C-T haplotype (both haplotypes contain the major allele at *rs2476601*) suggested a marginal protective effect independent of *rs2476601* (OR = 0.79, *P* = 0.05). However this effect was not replicated in the WTCCC sample set (OR = 1.13, *P* = 0.06) (Table 4). Consistent with this, conditional analysis on the combined NZ/WTCCC dataset did not support association of *rs12566340* independent of *rs2476601* (*P* = 0.76).

**Table 4:** *Rs2476601-rs12566340* haplotypic analysis.

Haplotype	Case (N, freq)	Cont (N, freq)	OR [95% CI]	*P*
**RA** 1				
C-C	1225 (0.716)2550 (0.721)3775 (0.719)	877 (0.784)4323 (0.771)5200 (0.773)	0.79 [0.63–1.00]1.13 [0.99–1.30]1.02 [0.91–1.14]	0.0480.0630.743
C-T	238 (0.139)381 (0.108)619 (0.118)	135 (0.121)733 (0.131)868 (0.129)	1.00 (reference)1.00 (reference)1.00 (reference)	---
T-C	12 (0.007)1 (0.000)13 (0.002)	1 (0.001)6 (0.001)7 (0.001)	---	---
T-T	237 (0.139)606 (0.171)844 (0.161)	105 (0.094)544 (0.097)649 (0.097)	---	---
**T1D**				
C-C	10287 (0.707)	10831 (0.776)	1.01 [0.94-1.09]	0.78
C-T	1685 (0.116)	1800 (0.129)	1.00 (reference)	-
T-C	35 (0.002)	36 (0.003)	-	-
T-T	2539 (0.175)	1289 (0.092)	-	-

1. Within each cell in the RA half, NZ data are top, WTCCC data middle and combined bottom.

Previously >150 SNPs in the *PTPN22* haplotype block and flanking 400 kb were genotyped in a British T1D case-control sample set with the aim of identifying putative T1D risk variants independent of *rs2476601* [13]. There was no evidence for allelic heterogeneity, with *rs2476601* remaining the best candidate for sole causal variant. However, none of the SNPs *rs12566340*, *rs7529353* or *rs11102691* was included in this analysis. We, therefore, genotyped *rs12566340* over the same T1D samples previously studied by Smyth et al [13], with no evidence for an effect at *rs12566340* independent of *rs2476601* (*P* = 0.85). Equality of risk conferred by the C-C haplotype in comparison to the C-T haplotype at *rs2476601-rs12566340* was also observed (Table 4; OR = 1.01 [0.94–1.09], *P* = 0.78), consistent with the absence of common allelic heterogeneity at *PTPN22* in T1D.

We also examined the rare T-C haplotype of *rs2476601-rs12566340*, testing for an effect on disease risk using the T-T haplotype as reference. In T1D there was evidence for an independent protective effect of the C allele (OR T-C = 0.48 [0.29–0.80], *P* = 0.004), but not in the combined NZ/WTCCC RA dataset (OR T-C = 1.43 [0.57–3.60]), *P* = 0.45).

Recently Steck et al [16] published evidence for a 6-marker protective haplotype in T1D, defined by markers in *C1orf178*, from a case versus control analysis of chromosomes containing only the Arg620 allele. The haplotype was tagged by the minor allele of *rs1539438* (A>G), which had been genotyped by Smyth et al [13] and its association with T1D shown to be dependent on *rs2476601*. We genotyped *rs1539438* in the NZ RA sample set and tested for association independent of *rs2476601* in the NZ samples, and in the publically-available WTCCC samples, with no evidence for independent association detected (*P* = 0.42 and 0.25, respectively).

## Discussion

The Trp620 allele (*rs2476601*) of the *PTPN22* SNP rs2476601 is strongly associated with both RA and T1D.[4], [13] Smyth et al [13] demonstrated that this allele explains the association of the *PTPN22* locus with T1D. They analysed 46 *PTPN22* SNPs, and 111 further SNPs from the *PTPN22* haplotype block and 400 kb flanking the haplotype block. Our approach in RA was different to that taken by Smyth et al [13], being driven by the findings of Carlton et al [9], beginning with studying the specific haplotypes that they reported that altered RA risk (haplotypes 4, 5 and 6–10). By generation of new data, and meta-analysis, we also found that these haplotypes influence the risk of RA, although with an opposing direction of effect in the case of haplotype 4. However, neither the haplotype-defining SNPs, nor *rs12566340* (haplotypes 4, 5, 6–10 converge to the major allele), were independently associated with RA. There was also no evidence for independent association of this SNP with T1D (neither *rs12566340*, nor any surrogate SNPs had previously been analysed by Smyth et al [13]). We conclude that it is unlikely that allelic heterogeneity at the *PTPN22* locus, driven by common variants, exists in RA. The published functional data [7], including correlations between TCR signalling and carriage of the different alleles of Arg620Trp strongly supports that this non-synonymous SNP is the causal variant. However, given the entire LD region has not yet been resequenced, there is still a possibility that as yet unidentified variant(s) could play a role in disease etiology.

It is important to note that our approach examined common Arg620Trp-independent variants. We did not address the question of whether or not there are rare Arg620Trp-independent variants, which are known to exist, in RA and SLE at least (Arg263Gln).[14], [15] There was evidence for inequality of risk for the rare T-C haplotype compared to the T-T haplotype in T1D, but this was not supported in the RA sample set. Further study of this rare haplotype will be difficult, owing to its scarcity. The 263Gln allele, which confers a protective effect in RA independent of Arg620Trp,[15] is nearly exclusively contained on haplotypes containing the major (620Arg) allele at *rs2476601* in Caucasians,[15] meaning it cannot explain any possible independent protective effect of the T-C haplotype.

Carlton et al [9] concluded that the Arg620Trp variant did not fully explain the association between *PTPN22* and RA. Using a haplotype method of analysis and conditional logistical regression they concluded that haplotype ‘4’ (tagged by the G allele of *rs3811021*) was primarily responsible for their observation that Arg620Trp did not fully explain the association of the *PTPN22* locus with RA. By testing for equality of risk between haplotypes containing *rs2476601* and *rs12566340*, we found no evidence for an RA risk effect independent of *rs2476601*, nor for any of the haplotype 4, 5 and 6–10 tagging SNPs. Our apparently conflicting findings need to be considered in light of the heterogeneity in association with RA of haplotype 4 between the USA European-ancestry samples studied by Carlton et al [9] (susceptible effect), and the British and European samples studied here (protective effect; note that the European population of NZ is predominantly derived from immigrants from Britain and Europe). Acknowledging that different statistical methods were used, it is possible that population-specific effects are obscuring investigation of an Arg620Trp-independent effect on RA risk at the PTPN22 locus. Certainly the LD between the haplotype ‘4’ and ‘5’-defining SNPs differs between the samples studied by Carlton et al [9] (r^2^∼0.4 [9]) and the WTCCC and HapMap CEU samples (r^2^∼0.1). It is unlikely that the difference in haplotype 4 results is caused by fluctuation in control frequencies, as was previously noted with respect to observation of a protective haplotype in a study of the *PTPN22* locus in Graves' disease [13], [17] – changes in both the control and case *rs3811201* frequencies are evident between the Carlton et al [9] and the newly analysed data (Table 3). Genotyping of *rs3811021*, *rs12566340*, *rs2476601*, *rs3789607* and other relevant SNPs over additional USA and British/European sample sets is warranted. The possibility of clinical heterogeneity between sample sets playing a role in the different outcomes of our and the Carlton et al studies should also not be overlooked. However, it is not possible to comprehensively consider this possibility presently, owing to the paucity of clinical data available for the relevant RA sample sets (refer to Samples and Methods and Carlton et al [9]).

## Methods

### Ethical Statement

Ethical approval for the NZ study was given by the MultiRegion and Lower South Ethics Committees, the UK London RA study by the Lewisham Hospital and Guy's and St. Thomas' Hospitals local research ethics committees, participants with T1D were enrolled under study protocols approved by the Institutional Review Board of each UK institution that contributed (see http://www.childhood-diabetes.org.uk/grid.shtml), and 1958 birth cohort controls for the T1D comparison by the London Multiregion Ethics Committee. All subjects gave written informed consent, or their parents/guardian for those considered too young to consent.

### Study subjects

Data from four separate RA case-control sample sets, all consisting of white European subjects, were analysed. All cases satisfied the 1987 American College of Rheumatology criteria for RA.[18]

1. The New Zealand (NZ) sample sets consisted of 860 RA patients and 564 controls. Cases were recruited from outpatient clinics in Auckland, Bay of Plenty, Wellington, Canterbury, Otago and Southland. Twenty-eight percent were male and 72% female. Of the RA patients for whom serologic data were available, 82% (571/697) were rheumatoid factor (RF) positive and 65% (303/465) were anti-cyclic citrullinated peptide (CCP) antibody positive. The NZ control samples (n = 557) consisted of healthy subjects recruited from Otago and Auckland.
2. The UK Wellcome Trust Case Control Consortium (WTCCC) patient group consisted of 1860 European individuals, of which 25% were male, and the control group (n = 2938) of healthy Caucasian individuals with a median age between 40–49 years, of whom 49% were male.[2]
3. The UK London sample set consisted of 235 RA patients recruited at Lewisham, and Guy's and St. Thomas's Hospitals, of whom 79.3% were female and 63.1% RF positive. 165 UK control samples were purchased from the European Collection of Cell Cultures (www.hpacultures.org.uk/collections/ecacc.jsp).
4. Published data from two sample sets were used. The Dutch sample set consisted of 667 cases and 286 controls recruited from Leiden[11] and the Norwegian sample set consisted of 861 cases and 559 controls recruited from Oslo[12]. Demographic and clinical data were not presented for either set of samples.

In T1D, 7,273 cases and 6,978 controls were genotyped for *rs2476601* and *rs12566340*. The affected individuals were recruited as part of the Juvenile Diabetes Research Foundation/Wellcome Trust Diabetes and Inflammation Laboratory's British case collection. Most individuals with T1D were <16 years of age at the time of collection (mean age at diagnosis 7.5 years, range 0.5 to 16 years) and all resided in Britain. The control samples were obtained from the British 1958 Birth cohort, an ongoing study of all people born in Great Britain during one week in 1958. All cases and controls were of self-reported white ethnic group and European ancestry.

### Genotyping and imputation

Subjects from the NZ and UK London sample sets were genotyped in this study using PCR-RFLP: *rs3789607* using primers GGCTGTTTTATTTCCCCTGT and GAGCTAGTTTGCTATCACTG that result in cleavage of the 160 bp product into 100/60 bp fragments using *aTaq*I; *rs12566340* using primers TGATCAATCTGATGGCAGTATATAGGACAA and CCTCAGTCATTTTTACCTTG that result in cleavage of the 210 bp product into 179/31 bp fragments using *Tsp*509I; and *rs12144309* using primers ATGGCACCTCAGATGCATTA and AGTATTTACATATTTAATCCACCTGGAATC that result in cleavage of the 176 bp product in 145/31 bp fragments using *aTaq*I. *rs1539438* was genotyped over the NZ samples by Taqman (Applied Biosystems) using probe C_1900118_10 and primers GTAATTTTATTAAGAAATACTTCCT[C/T] and GACTTCTTAGGTCCTGCACATGGTA.

Subjects from the T1D sample set were genotyped with TaqMan, which was carried out in accordance with the manufacturers' protocols. All genotyping data were scored blind to case-control status; TaqMan genotyping was double scored by a second operator to minimize error.

Access to genotype data was granted by the WTCCC (www.wtccc.org.uk). Genotypes for *rs12144309* and *rs12566340* were imputed using *IMPUTE v0.2.0* [19] with default parameters, a 10 Mb window centred on the PTPN22 locus and called using a quality threshold of 0.9.

### Data analysis

All genotype data were checked for deviation from Hardy-Weinberg equilibrium using http://ihg.gsf.de/cgi-bin/hw/hwa1.pl. The NZ control samples exhibited a small deviation from Hardy-Weinberg equilibrium (HWE) for markers *rs12566340* and *rs12144309* (*P* = 0.009 and 0.02, respectively). 20% of genotypes for each of *rs12566340* and *rs12143309* were consequently repeated, with 100% concordance for both. SHEsis [20] was used to generate basic summary statistics (allelic and genotypic *P* values by Fisher's chi-square, testing for deviation from HWE). UNPHASED[21] was used to test for equality of risks between haplotypes and to test for association at one marker conditional upon a second. STATA 8.0 was used to calculate Mantel-Haenszel (M-H) pooled ORs and test for heterogeneity between datasets, using a fixed effects model in the absence, and a random effects model in the presence, of heterogeneity. The power to detect a putative independent causal effect of weak magnitude (OR = 1.2/0.83, alpha = 0.01) owing to *rs12566340* was 97% for RA and 100% for T1D.

## References

[1] Steer S, Abkevich V, Gutin A, Cordell HJ, Gendall KL (2007). Genomic DNA pooling for whole genome association scans in complex disease: Empirical demonstration of efficacy in rheumatoid arthritis.. Genes Immun.

[2] The Wellcome Trust Case Control Consortium (2007). Genome-wide association study of 14,000 cases of seven common diseases and 3,000 shared controls.. Nature.

[3] Plenge RM, Seielstad M, Padyukov L, Lee AT, Remmers EF (2007). TRAF1-C5 as a risk locus for rheumatoid arthritis–a genomewide study.. N Engl J Med.

[4] Lee YH, Rho YH, Choi SJ, Ji JD, Song GG (2007). The PTPN22 C1858T functional polymorphism and autoimmune diseases–a meta-analysis.. Rheumatology.

[5] Kawasaki E, Awata T, Ikegami H, Kobayashi T, Maruyama T (2006). Systematic search for single nucleotide polymorphisms in a lymphoid tyrosine phosphatase gene (PTPN22): association between a promoter polymorphism and type 1 diabetes in Asian populations.. Am J Med Genet A.

[6] Wu J, Katrekar A, Honigberg LA, Smith AM, Conn MT (2006). Identification of substrates of human protein-tyrosine phosphatase PTPN22.. J Biol Chem.

[7] Vang T, Congia M, Macis MD, Musumeci L, Orrú V (2005). Autoimmune-associated lymphoid tyrosine phosphatase is a gain-of-function variant.. Nat Genet.

[8] Arechiga AF, Habib T, He Y, Zhand X, Zhang Z-Y (2009). The PTPN22 allelic variant associated with autoimmunity impairs B cell signaling.. J Immunol.

[9] Carlton VE, Hu X, Chokkalingam AP, Schrodi SJ, Brandon R, Alexander HC (2005). PTPN22 genetic variation: evidence for multiple variants associated with rheumatoid arthritis.. Am J Hum Genet.

[10] Hinks A, Eyre S, Barton A, Thomson W, Worthington J (2007). Investigation of genetic variation across the protein tyrosine phosphatase gene in patients with rheumatoid arthritis in the UK.. Ann Rheum Dis.

[11] Wesoly J, Hu X, Thabet MM, Chang M, Uh H (2007). The Trp620 allele is the PTPN22 genetic variant conferring susceptibility to RA in a Dutch population.. Rheumatology (Oxford).

[12] Viken MK, Olsson M, Flam ST, Forre O, Kvien TK (2007). The *PTPN22* promoter polymorphism -1123G>C association cannot be distinguished from the 1858C>T association in a Norwegian rheumatoid arthritis material.. Tissue Antigens.

[13] Smyth DJ, Cooper JD, Howson JM, Walker NM, Plagnol V (2008). PTPN22 Trp620 explains the association of chromosome 1p13 with type 1 diabetes and shows a statistical interaction with HLA class II genotypes.. Diabetes.

[14] Orrú V, Tsai SJ, Rueda B, Fiorillo E, Stanford SM (2009). A loss-of-function variant of PTPN22 is associated with reduced risk of systemic lupus erythematosus.. Hum Mol Genet.

[15] Rodrígeuz- Rodrígeuz L, Wan Taib WR, Topless R, Steer S (2010). The PTPN22 R263Q polymorphism is a risk factor for rheumatoid arthritis in Caucasian case-control samples..

[16] Steck AK, Baschal EE, Jasinski JM, Boehm BO, Bottini N rs2476601 T allele (R620W) defines high-risk PTPN22 type I diabetes-associated haplotypes with preliminary evidence for an additional protective haplotype.. Genes Immun.

[17] Heward JM, Brand OJ, Barrett JC, Carr-Smith JD, Franklyn JA, Gough SC (2007). Association of PTPN22 haplotypes with Graves' disease.. J Clin Endocrinol Metab.

[18] Arnett FC, Edworthy SM, Bloch DA, McShane DJ, Fries JF (1988). The American Rheumatism Association 1987 revised criteria for the classification of rheumatoid arthritis.. Arthritis Rheum.

[19] Marchini J, Howie B, Myers S, McVean G, Donnelly P (2007). A new multipoint method for genome-wide association studies by imputation of genotypes.. Nat Genet.

[20] Shi YY, He L (2005). SHEsis, a powerful software platform for analyses of linkage disequilibrium, haplotype construction, and genetic association at polymorphism loci.. Cell Res.

[21] Dudbridge F (2003). Pedigree disequilibrium tests for multilocus haplotypes.. Genet Epidemiol.

